# Predicting the Effect of Surface Texture on the Qualitative Form of Prehension

**DOI:** 10.1371/journal.pone.0032770

**Published:** 2012-03-05

**Authors:** Ian John Flatters, Loanne Otten, Anna Witvliet, Brian Henson, Raymond John Holt, Pete Culmer, Geoffrey Parker Bingham, Richard McGilchrist Wilkie, Mark Mon-Williams

**Affiliations:** 1 Institute of Psychological Sciences, University of Leeds, Leeds, West Yorkshire, United Kingdom; 2 School of Mechanical Engineering, University of Leeds, Leeds, West Yorkshire, United Kingdom; 3 Pedagogische Wetenschappen en Onderwijskunde, Radboud Universiteit Nijmegen, The Netherlands; 4 Department of Brain and Psychological Sciences, Indiana University, Bloomington, Indiana, United States of America; Bielefeld University, Germany

## Abstract

Reach-to-grasp movements change quantitatively in a lawful (i.e. predictable) manner with changes in object properties. We explored whether altering object texture would produce qualitative changes in the form of the precontact movement patterns. Twelve participants reached to lift objects from a tabletop. Nine objects were produced, each with one of three grip surface textures (high-friction, medium-friction and low-friction) and one of three widths (50 mm, 70 mm and 90 mm). Each object was placed at three distances (100 mm, 300 mm and 500 mm), representing a total of 27 trial conditions. We observed two distinct movement patterns across all trials—participants either: (i) brought their arm to a stop, secured the object and lifted it from the tabletop; or (ii) grasped the object ‘on-the-fly’, so it was secured in the hand while the arm was moving. A majority of grasps were on-the-fly when the texture was high-friction and none when the object was low-friction, with medium-friction producing an intermediate proportion. Previous research has shown that the probability of on-the-fly behaviour is a function of grasp surface accuracy constraints. A finger friction rig was used to calculate the coefficients of friction for the objects and these calculations showed that the area available for a stable grasp (the ‘functional grasp surface size’) increased with surface friction coefficient. Thus, knowledge of functional grasp surface size is required to predict the probability of observing a given qualitative form of grasping in human prehensile behaviour.

## Introduction

Most humans demonstrate an exquisite ability to manipulate objects with their hands. Expert manual interaction with an object requires the actor to move their hand to the object of interest (the precontact phase) and then apply the appropriate fingertip forces in order to manipulate the object (the contact phase). In the precontact phase, the geometric properties of the object constrain the trajectory of the grasp such that the digits align with the object surface [1], [2]. In the contact phase, the physical properties of the object determine the fingertip forces required for manipulation.

In line with this, it has been shown that the textural properties of objects influence the contact phase of prehension [3]. Contact with an object provides haptic information regarding its textural properties and this information is known to be used in programming the appropriate fingertip forces [4]. Nevertheless, vision can provide useful information regarding object properties before the time of contact. Visual information can therefore be used to programme forces in advance, on the basis of memorised textural properties (acquired over the lifespan and/or from immediately preceding object interactions). Forsberg and colleagues have shown that visual information is used in this way, with the properties of an object influencing the fingertip forces programmed in advance of contact [4].

The fact that texture influences the advance programming of fingertip forces implies that an object's texture might affect the precontact phase of the movement. This is particularly important as the influence of texture on the precontact phase of prehension has clinical applications, with a number of older adults experiencing difficulties when handling everyday items (e.g. a hot cup of tea or a saucepan handle). There has been remarkably little investigation of this topic. Weir et al. [5] reported that texture had no impact upon the duration of the precontact phase but low-friction surfaces increased the time that participants spent generating fingertip forces before the object was lifted. In contrast, Fikes et al. [6] did find an effect of texture on the precontact phase, with participants taking longer to move their hand to a low-friction object. Thus there is some empirical evidence that quantitative changes in prehension occur as a function of surface texture. The question of whether surface texture influences the qualitative form of the precontact movement patterns, however, remains unanswered. This question is of particular interest because it has both practical and theoretical implications. If different textures (and their visual appearances) produce different qualitative patterns then, at a practical level, engineers can determine whether different surfaces have the potential to elicit safer behaviour (e.g. can kitchen utensils be made safer for older adults to reach-and-grasp?).

The question is also pertinent to the theoretical issue of action selection: what makes us select one movement pattern rather than another when interacting with objects that afford multiple options? Modern theoretical accounts of motor control suggest that actions are controlled via ‘inverse models’ – neural circuits that have become reinforced because their activation produces the desired movement pattern when triggered by a given input stimulus [7]. It is thought that multiple inverse models are housed within the brain, with many of these models sharing common neural architecture. In this conceptual framework, the acquisition of a new skill occurs through the modification of an existing neural circuit, producing a new internal model that is precisely tuned to specific environmental conditions. This postulated mechanism allows the acquisition of complex skills through the merger of a series of discrete movements that achieve particular goals. The resulting ‘higher-order’ behaviour might result in ‘lower-order’ movements unfolding concurrently or in rapid sequential order. This can be conceived as a process where ‘higher-order’ models recruit ‘lower-level’ models (in the same way that sub-routines are called within a complex computer programme). The notion of multiple inverse models suggests that a small environmental change (e.g. a different surface texture) might be sufficient to trigger a different higher-order inverse model and thus elicit a qualitatively different action - despite the task appearing to require the same class of movement. There have been few empirical investigations into this topic, hence our interest in the issue of whether surface texture can influence the qualitative prehension movement pattern.

**Figure 1:**
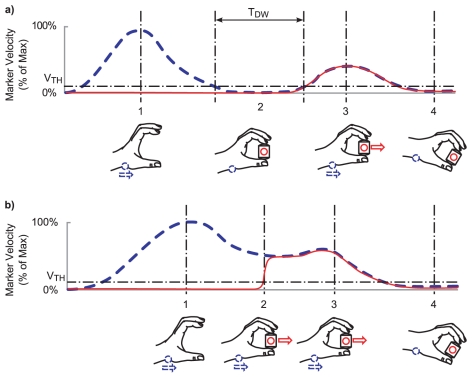
Kinematic profiles for stop and ‘on-the-fly’ prehension movements. *Upper* A velocity profile typical of a stop movement: 1, the hand is in the transport phase with the wrist IRED reaching peak velocity. 2, as the hand and fingers approach the object the hand velocity drops below the threshold velocity (V_TH_) and remains below threshold velocity or stops for a period (T_DW_). 3, upon successful application of the grip, both the wrist and object markers move in unison as part of a second distinct movement. 4, movement complete – hand and object velocity tends to zero. *Lower* A velocity profile typical of a ‘fly-through’ movement: 1, the hand is in transport phase toward the object. 2, as the fingers contact the object, the wrist IRED velocity is maintained above the threshold velocity (V_TH_) as the object is gripped. 3, the hand and object continue to move in unison while the wrist IRED velocity remains above the threshold velocity. 4, movement complete, hand and object velocity tends to zero.

**Figure 2:**
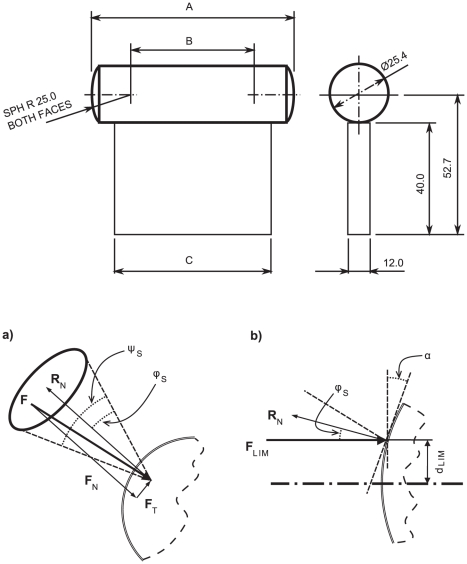
Object geometric properties friction-dependant functional grip area. *Upper* Geometric variation in stimulus sizes: Grip surface width ‘A’, the distance between the spherical surface centre-points ‘B’ and support base width ‘C’ were varied as discussed in the Method section. *Lower a)* Manually securing an object requires the frictional force to be greater than the tangential component of object weight at the interface between fingertip and object. A curved surface results in a normal reaction force direction (R_N_) unique to the point at which the object is grasped. Fearing [14] demonstrated that, for a stable grasp, the grip conditions should satisfy: tan^−1^|F_t_|/F_n_<tan^−1^μ or μF_n_>|F_t_|. For a stable lift, fingertip force should be applied within an angle of φ_s_ relative to the normal reaction force (R_N_), where: φ_s_ = tan^−1^μ_s_. Extending this relationship in the direction of all tangential friction force directions generates a cone of friction of half-angle φ_s_ and cone angle ψ where: ψ = 2 φ_s_. *b)* As force is applied to the curved surface at a distance d_LIM_ from the centreline of the radius, then the force is at an angle α to the surface normal. When α = φ_s_ the force lies at the limit of the cone of friction. An increase in d results in the force lying outside the cone of friction and unstable grasp. Thus φ_s_, and d_LIM_ are linked to the coefficient of static friction μ_s_ such that an increase in μ_s_ extends the functional area which can be grasped to achieve a stable grasp.

Mon-Williams and Bingham [8] have shown that two distinct movement patterns can emerge when participants are asked to reach-and-grasp an object and lift it off a tabletop (see Figure 1). In some cases, participants stop their arm moving forward before the fingers make contact with the object, adjust finger position and then grasp and lift (so-called ‘stop’ movements). In other cases, participants contact the object whilst the hand is still moving (so-called ‘on-the-fly’ movements). If the safety margins of the task decrease (e.g. by making the object wider and closer to the maximum grasp aperture) then the proportion of on-the-fly movements also decreases. This observation suggests that the probability of observing a particular movement pattern is affected by the margins of safety. On these grounds, we hypothesised that changes in an object's surface texture might alter the proportion of on-the-fly movements, because altering texture affects the safety margins (see Figure 2, Lower Panel).

In order to explore the manner in which humans interact with objects of different textural properties, we asked participants to reach-to-grasp and lift objects from a tabletop while experimentally manipulating object width, distance and surface texture. We expected that changes in the distance of the object would produce the normal lawful changes in the reach kinematics (higher peak speeds and longer durations for further distances). More importantly, Mon-Williams and Bingham's [8] findings led us to predict that decreasing the surface friction would decrease the proportion of on-the-fly movements.

## Methods

Twelve unpaid participants from the University of Leeds were recruited (7 female; age mean 27.7 years, age range 20.5–47.1 years; 11 reported right hand preference). All participants had normal or corrected-to-normal vision and no history of neurological deficit. Maximum pinch grip aperture was measured for each participant using a ruler (mean 15.8 cm, range 13.0–21.0 cm). All participants provided informed consent prior to inclusion in the study. The study was approved by a University ethics committee and was performed in accordance with the ethical standards laid down in the Declaration of Helsinki.

The stimuli were manufactured by mounting a plastic (nylon, black) cylinder (25.4 mm diameter) on a wooden block (Figure 2, Upper Panel). The ends of each plastic cylinder were machined to a 25 mm radius. Participants grasped along the long axis of the cylinder between the thumb and index finger. Three object widths were used (dimension A: 50, 70 and 90 mm, Figure 2, Upper Panel) while the distance between spherical centre-points of the grip surfaces (dimension B: 0, 20 and 40 mm, Figure 2, Upper Panel) and the wooden mounting block width (dimension C: 33, 53 and 73 mm, Figure 2, Upper Panel) varied proportionally to the object width. For each of the three object widths, there were three different surface textures applied to the grasp surfaces, such that three distinct coefficients of friction would be generated: High (μ_H_), Medium (μ_M_) and Low (μ_L_). The high-friction surface was generated by sticking coarse-grade sandpaper (Aluminium Oxide, P50) to the grasp surfaces. The medium-friction surface was the untreated machined plastic. The low-friction condition was achieved through the application of petroleum jelly (Vaseline®, Unilever) with a soft-bristled brush to the participant's fingertips and the grasp surfaces of the machined plastic stimulus (application was repeated on alternate trials).

To confirm that manipulation of the coefficient of friction was occurring at the fingertip interface, the coefficients of friction (μ_H_, μ_M_ and μ_L_) were calculated experimentally using apparatus developed by Shao, et al. [9]. Each sample was placed on a two-axis load cell and a vertical load of approximately 1N was applied (Y-axis) through the silicone fingertip onto the sample. A horizontal displacement of the fingertip was applied at 10 mm/s (X-axis) until the fingertip was clear of the sample. Force data were sampled at 1000 Hz in the X and Y components. Each test was repeated three times. The data were filtered using a dual-pass Butterworth second order filter with a cut-off frequency of 16 Hz (equivalent to a fourth order zero phase lag filter of 10 Hz). The coefficient of static friction was calculated by dividing the maximum value of horizontal force by the component of vertical force at the corresponding time point.

To ensure a consistent starting position, the participants pinched a raised origin marker positioned 100 mm from the front edge of the study table prior to the start of each trial. The objects were placed at distances of 100, 300 and 500 mm beyond the origin point, in line with the midline of the participant. Participants were instructed to reach and grasp the object as quickly and as accurately as possible between the pads of the forefinger and thumb, lift the stimulus from the table and hold it in a static raised position until told to lower the object to the table and return to the start position in preparation for the next trial. Participants were instructed to begin movement when they heard a verbal “go” command at the end of a verbal countdown, i.e. “three, two, one, go”. Data acquisition was initiated when the participant was still pinching the origin point (at the count of “one”), and the hold phase of the movement lasted between 0.5 s and 1 s.

The factors of object width and distance were presented in a pseudo-randomised order. Participants were blocked and counterbalanced on the factor of surface friction coefficient. The three object widths, three object distances and three coefficients of friction represented 27 conditions, each of which was repeated 10 times, resulting in a total of 270 trials. The test session typically lasted 1 hour. Trial repetition criteria included: (i) Failure to grip the stimuli on the instructed surface; (ii) Inability to achieve stable, static grip of the stimuli; (iii) Knocking the stimuli over; (iv) Dropping the object prior to, or shortly after, the verbal return command. Following failure of a trial, the condition under which failure occurred was recorded and the participant returned to the origin and repeated the trial. In the low-friction object condition, 4.1% of trials required repetition compared to a repetition rate of 2.4% across all trials. This procedure ensured that 10 trials for each condition were completed.

Kinematic data acquisition was performed using an Optotrak 3020 motion tracking system (Northern Digital, Ontario, Canada). The positions of four Infra Red Emitting Diodes (IREDs) were acquired at 100 Hz for three seconds for the high-friction and medium-friction conditions and for four seconds on the low-friction conditions (because the low-friction surface took longer to pick up). The first two markers were attached to the reaching hand at the index finger (distal medial corner of the finger) and the thumb (distal lateral corner of the thumb). These markers were used to measure grip aperture. The third marker was placed on the styloid process of the wrist to provide an independent measure of hand movement. A fourth marker was placed on the wooden block of the stimuli facing away from the participant to identify when the object was lifted off the tabletop. All data were filtered using a dual-pass Butterworth second order filter with a cut-off frequency of 16 Hz (equivalent to a fourth order zero phase lag filter of 10 Hz). The distance between the thumb and index finger IREDs (the aperture) was then computed. Following this operation, the speed of the wrist IRED and the aperture was computed and the onset and offset of movement together with the peak speed was estimated using standard velocity threshold and peak picking algorithms (threshold for movement onset and offset was 50 mm/s as per Munro et al. [10]). The criterion for onset of a reach was wrist velocity exceeding 50 mm/s. The criterion for cessation of reach movement was wrist velocity falling below 50 mm/s. The deceleration phase was defined as the time between peak speed and the offset of reach movement. The object's ‘time-to-lift’ was designated at the point when the fourth IRED's velocity exceeded 50 mm/s. The critical issue was whether movements were ‘stop’ or ‘on-the-fly’. Movements were classified as ‘stop’ if there was a temporal gap between the cessation of wrist movement and the onset of movement of the object. Movements were classified as ‘on-the-fly’ if the wrist velocity was maintained above the threshold velocity from the onset of wrist movement to the onset of object movement. This procedure allowed a simple objective classification of the different movement types (see Figure 1). Visual inspection of the trials confirmed that this objective classification was rational – there was a clear bifurcation whereby the hand would either clearly stop before the lift or the object was grasped whilst the hand was still travelling above the threshold velocity.

The mean value across the 10 trials for each dependent variable of interest for each individual participant was entered into a 3 (Distance)×3 (Width)×3 (Surface Texture) repeated measures ANOVA (a separate ANOVA for each dependent variable of interest).

## Results

### “On-the-fly” Movements

**Figure 3:**
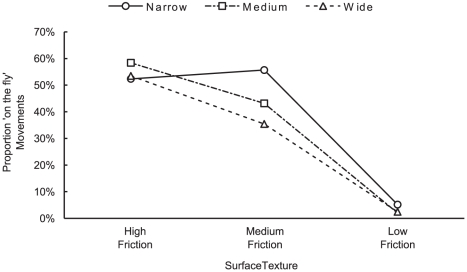
Proportion of ‘on-the-fly’ movements as a function of surface texture. The mean coefficient of static friction was 1.31, 0.76 and 0.44 for the high, medium and low friction object surface textures respectively (see Methods).

The proportion of on-the-fly movements was affected by the grip surface (F(2,22) = 20.15, p<0.01) and object width (F(2,22) = 8.60, p<0.01) (Figure 3), with a statistically reliable interaction between the two (F(2,22) = 4.34, p<0.05, ε = 0.77). The narrow width object produced a similar proportion of on-the-fly movements in the medium and high friction conditions. It is not clear why this was the case, but the clear difference between these conditions and the low-friction target is the critical finding. We found no effect of distance (F(2,22) = 0.91, p = 0.41), nor interactions of distance with width or surface texture. We explored the data to determine whether stop movements reliably followed a failed trial or whether ‘hysteresis’ could be observed in the data (where one trial influences the next) but we were unable to identify any discernible pattern.

The peak speed of the movement was affected by object distance (F(2,22) = 241.88, p<0.001, ε = 0.518) but not by width or texture or interactions. Increased reach distance caused a longer Movement Time (MT) (F(2,22) = 36.27, p<0.01, ε = 0.77). There was a two way interaction between texture and object width, with MT increasing as the surface friction decreased and these effects being more pronounced when the object was wider (F(4,44) = 35.33, p<0.01, ε = 0.76). The MT increases could be explained through a prolonged deceleration phase, so there was a two way interaction between texture and object width, with deceleration time increasing as the surface friction decreased and these effects being more pronounced when the object was wider (F(4,44) = 7.46, p<0.01, ε = 0.41).

## Discussion

Humans are complex systems and human behaviour is notoriously difficult to predict. But behaviour is not random and invariant patterns can be found in tasks such as reaching-to-grasp objects [11]. For example, the duration of the movement is lawfully related to the distance of the object to be grasped [12]. Thus, it is possible to predict the quantitative relationship between duration and object distance for a given individual carrying out a particular prehensile task [13]. The present study explored whether we might find similar invariant patterns in the *qualitative* form of reach-to-grasp movements. Mon-Williams and Bingham [8] have shown previously that the instruction to reach, grasp and lift an object from a tabletop produces two distinct movement patterns. In some cases, the participants move their hand to the object, stop, secure a grasp, then lift the object upwards. In other cases, participants grasp the object ‘on-the-fly’ such that the arm does not stop moving while the object is secured between the digits. We hypothesised that the proportion of these different movement patterns would be affected by the surface texture of the objects being grasped. In order to test this hypothesis we used three textures and studied whether the surface influenced the proportion of on-the-fly movements. The data showed unambiguously that surface texture altered the way in which participants interacted with the objects. The low-friction surface almost invariably caused participants to stop their arm moving forward before securing the object between the index finger and thumb, and then lifting the object from the tabletop. Thus, the behaviour was sequential in nature, with the reach, grasp and lift component occupying its own temporal space. In contrast, the reach, grasp and lift components were frequently merged into a single ‘higher-order’ behaviour with a high-friction surface texture.

The findings indicate that predicting the mode of human prehension requires knowledge of the object surface texture. In the case of the low-friction object, one can predict with reasonable certainty that individuals within the age range of 20–50 years will not show on-the-fly behaviour under these task conditions. The situation is more interesting with the high-friction surface texture. On average, on-the-fly behaviour is most likely to be seen over a series of repeated lifts, but it is not possible to be certain on any given trial whether the participant will stop before grasping. In the case of the medium-friction surface, it is close to chance as to whether the participant will stop or fly through.

It is of note that the peak speed of the movement was unaffected by the texture of the objects. The modular organisation of movements via multiple inverse models (as outlined in the introduction) is consistent with this finding. Multiple inverse models allow the system to acquire complex skills by combining ‘lower-order’ actions in countless ways and provide flexibility for tailoring behaviour to precise environmental conditions. In the present example, the goal directed behaviour can be conceived as three separate actions (‘reach’, ‘grasp’ and ‘lift’) underpinned by internal models that can be organised to unfold sequentially (the higher-order ‘stop’ behaviour) or concurrently (‘on-the-fly’). Such organisation is efficient as it allows recruitment of similar neural circuits (and thereby produces movements that show great similarity in the initial stages). It seems reasonable to assume that ‘stop’ reaches to the low-friction object were selected from the outset (given that this behaviour was almost inevitably observed on every trial). In the high-friction case, it is not possible for us to determine what action was initially selected. Mon-Williams and Bingham [8] have shown previously that participants can switch from ‘on-the-fly’ to ‘stop’ patterns as the movement unfolds in response to online feedback. This suggests that it might be possible after the event to identify factors that influence the qualitative movement pattern observed, but prediction before the trial starts must be probabilistic in nature.

The results from the rough object (where some movements were on-the-fly and some were stop) reveal the inherently probabilistic nature of predicting human behaviour. Nevertheless, an understanding of the probabilities of observing different behaviours allows the scientist to better predict the outcome of a given reach-to-grasp task. Weir et al. [5] and Fikes et al. [6] have previously reported a quantitative effect of texture on the precontact phase of prehension, with participants taking longer to move their hand to a low-friction object. The data from the current study support these previous observations. It follows that a complete description of reach-to-grasp behaviours requires knowledge of surface texture if the qualitative and quantitative form of the movement is to be predicted, though predictions about this human behaviour remain probabilistic in nature (especially, as observed by Neils Bohr, if the predictions are made in advance).
